# Adverse Metal Reaction in a Case of Metal-on-Polyethylene Hip Arthroplasty

**DOI:** 10.7759/cureus.60810

**Published:** 2024-05-21

**Authors:** Nikhil Singh, Abhiram Awasthi, Gajanan Pisulkar, Shounak Taywade, Nitin Samal

**Affiliations:** 1 Department of Orthopaedic Surgery, Jawaharlal Nehru Medical College, Wardha, IND

**Keywords:** metallurgy, revision joint replacement, hip joint, adverse reaction to metal debris, total hip replacement (thr)

## Abstract

Soft tissue inflammatory responses to metal debris from prostheses, categorised as adverse reactions to metal debris (ARMD), are frequent complications of total hip arthroplasty (THA) and often result in implant failure. Introducing modular implant designs in modern orthopaedics has brought benefits to total hip replacements but has also increased patients' susceptibility to corrosion-related risks. ARMD can develop from various metal articulating surfaces, including ceramic-on-polyethylene (CoP), ceramic-on-ceramic (CoC), metal-on-metal (MoM), and metal-on-polyethylene (MoP) configurations. In this case study, a 68-year-old male who underwent a MoP implant for osteoarthritis of the right hip 16 years ago presented with pain and difficulty walking, exacerbated over the past three months. Clinical examination revealed tenderness around the implant and a limited range of motion. Imaging studies, including X-rays and ultrasound-guided aspiration, coupled with normal serum and urinary cobalt (Co) and chromium (Cr) levels, confirmed the diagnosis of ARMD. Given the severity of symptoms and radiographic findings, surgical intervention was warranted, leading to a two-stage revision with implant augmentation using a Burch-Schneider cage. Three months post operation, the patient experienced significant improvements in pain levels, range of motion (ROM), and hip function. This case underscores the importance of vigilant surveillance for ARMD in patients undergoing non-MoM THA, even years post surgery. Prompt recognition and management of ARMD are crucial to mitigate the risk of long-term complications and optimise patient outcomes. Further research is needed to understand the risk factors and mechanisms underlying ARMD in MoP THA, aiding in developing preventive strategies and refined treatment protocols.

## Introduction

Osteoarthritis, commonly referred to as OA, is a prevalent and disabling joint condition impacting millions worldwide. It is the most widespread form of arthritis, characterised by gradual erosion of the articular cartilage and the underlying bone structure. Numerous factors contribute to its onset, including age, joint injury, obesity, genetic predisposition, and irregular joint structure. While a complete cure remains elusive, diverse treatment approaches exist to alleviate symptoms and enhance the well-being of those affected. These remedies encompass pain management medications, physical therapy, lifestyle adjustments, assistive tools, and, in severe instances, surgical interventions like joint replacements.

The advent of low-friction arthroplasty by Sir John Charnley revolutionised orthopaedic surgery, particularly in managing hip joint ailments such as osteoarthritis. This groundbreaking concept involves substituting a damaged hip joint with artificial components to minimise wear and enable smooth, frictionless motion. Charnley's pioneering method surpassed joint replacement techniques by introducing a metal femoral component fitted into the femur and a high-density polyethylene acetabular cup placed within the acetabulum. This strategic combination drastically reduced articular surface friction, fostering more natural joint movement and enhancing durability. While metal-on-polyethylene (MoP) plain bearings currently dominate prosthetic implantation, recent attention has shifted to metal-on-metal (MoM) bearings, prompted by findings of higher-than-anticipated revision rates.

Complications from metal wear are observed in patients undergoing joint replacement surgeries involving metal components. These issues result from the erosion of metal implants, releasing metal particles and ions into surrounding tissues and the bloodstream. This poses a significant concern, especially in MoM joints, commonly found in hip replacements, where both ball and socket components are metallic. During orthopaedic procedures, metal deposition can trigger inflammatory reactions in the body, ranging from mild discomfort and pain to more severe consequences such as tissue damage, implant instability, and systemic effects due to exposure to metal ions. Although uncommon, the development of an adverse local tissue reaction (ALTR), an immune response to metal debris during total hip arthroplasty (THA), remains a noteworthy complication [1].

## Case presentation

A 68-year-old male manual worker by profession came to our hospital with chief complaints of pain and difficulty in walking for one year, which has increased in the past three months. He underwent total hip replacement (THR) on his right side 16 years back for osteoarthritis of the right hip. An uncemented total hip prosthesis was implanted with a posterior approach. During the examination, no abnormal alterations were observed in the surgical scar. However, the swelling was evident on the right thigh, accompanied by a restricted motion at the right hip (Table 1).

**Table 1:** Range of motion at the right hip

Hip range of motion	
Flexion	<90°
Extension	<20°
Abduction	<40°
Adduction	<20°

**Figure 1:**
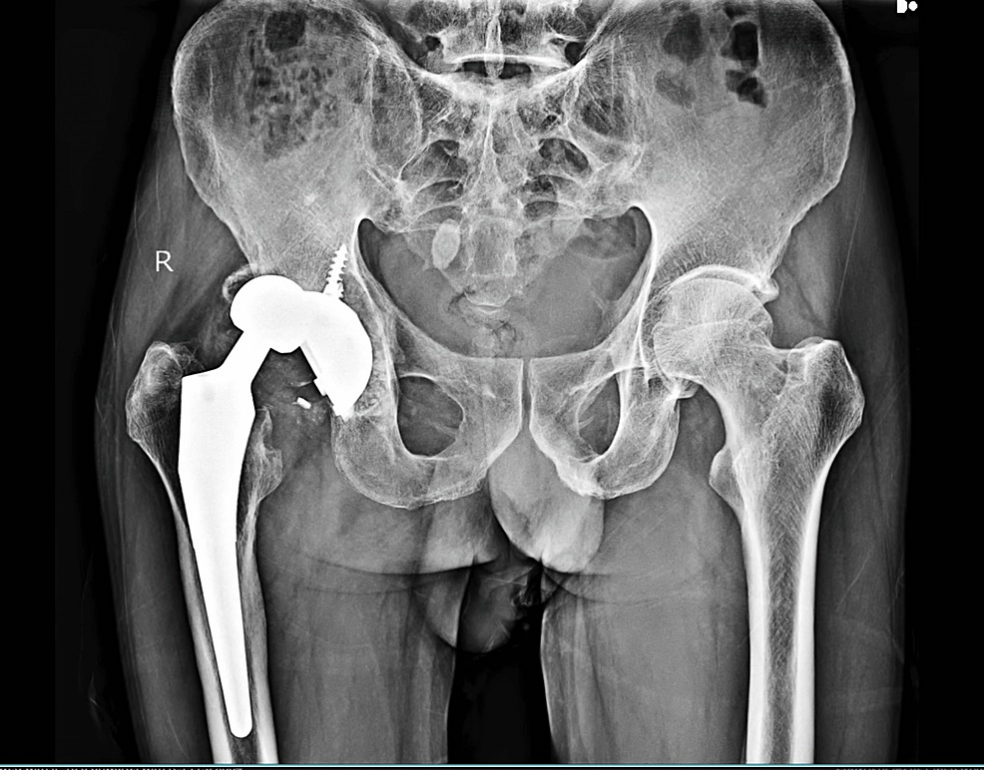
Pre-operative X-ray of the pelvis with both hips (anteroposterior view)

The pain assessed using the Visual Analogue Scale (VAS) was rated 6. X-ray of the pelvis with both hips showed femoral head dislocation articulating with the superior acetabulum cup with no evidence of fractures, broken acetabular liner, lysis around the acetabulum cup, bone loss in the posterosuperior part of the acetabulum, and well-fixed femoral stem (Figure 1).

The laboratory results of the patient are presented in Table 2.

**Table 2:** Preoperative investigations

Investigation	Pre-operative values	Normal range of value
Haemoglobin (Hb)	11.7 g/dL	12-16 g/dL
White cell count	8300	4.5-11.0 x 109/L
Platelet count	2.36 lakh	150-400 × 10^3^
C-reactive protein (CRP)	40.0 mg/L	<5 mg/L
Cobalt urine	7 ug/L	<2.0 ug/L
Chromium urine	8 ug/L	<2.0 ug/L
Cobalt blood	12 ug/L	<1.0 ug/L
Chromium blood	10 ug/L	<1.0 ug/L

**Figure 2:**
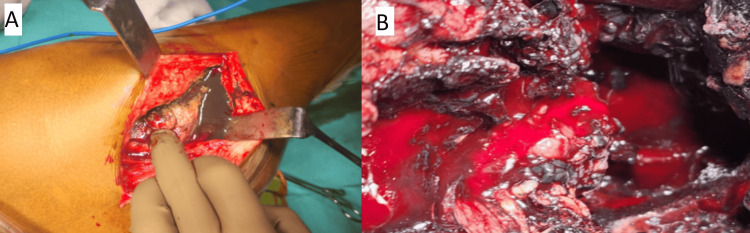
Intra-operative picture of metal debris around the hip joint (A) Black-coloured metal debris. (B) Stained soft tissue around the hip.

Ultrasound-guided aspiration was performed on the right hip, and the aspirated fluid was sent for culture, analysis of fluid characteristics, and cellular examination. Due to the severity of symptoms and radiographic findings, surgical intervention was deemed necessary, prompting the planning of a two-stage revision procedure with consideration for infection. The initial stage was conducted via the previous posterior incision. Upon incision of the deep fascia, the presence of black-coloured metal debris surrounding the femoral head, trunnion, stem, and adjacent areas confirmed the diagnosis of metallosis (Figure 2).

**Figure 3:**
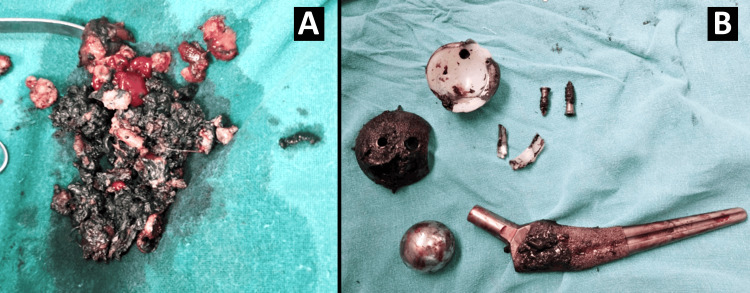
Black colour stained soft tissue and prosthetic components (A) Black colour stained soft tissue. (B) Prosthetic components removed.

Swab and tissue samples were collected for microbiological and histological assessment. All the metal implants and broken polyethylene liner fragments were removed from the surrounding soft tissues. The retrieved acetabular components were severely abraded with a broken polyethylene liner with medial bone loss in the acetabulum and thin margins of acetabulum; the Biomet Taperloc stem was not loose, and removed with drilling long k wire and use of small osteotomes with femoral head 32 intact. There was little corrosion at the neck taper junction (Figure 3).

**Figure 4:**
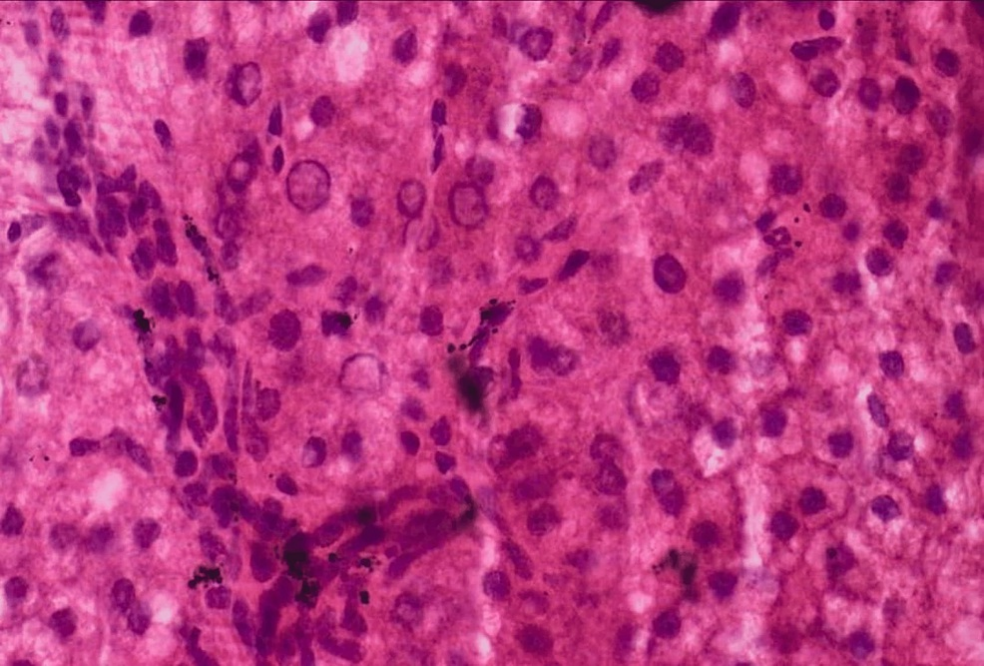
Black deposit can be seen with macrophages in hematoxylin and eosin (H&E)-stained sample

Thorough debridement was done, and the joint was irrigated with 6 L of normal saline, Betadine, and hydrogen peroxide. Immediately after the surgery, the operated limb was kept in skeletal traction. The patient was started with empiric antibiotic therapy. Cultures were negative in microbiological assessment. The histological examination confirmed the adverse reaction to metal debris (ARMD) diagnosis, describing multinucleated giant cells formed by the fusion of macrophages in response to foreign material with an iron deposit (Figure 4).

**Figure 5:**
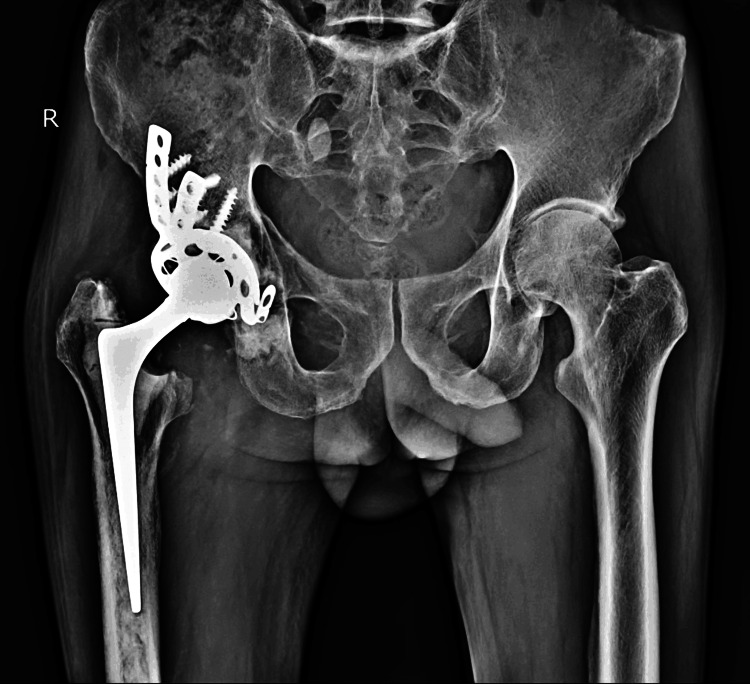
Post-operative X-ray of the pelvis with both hips (anteroposterior view)

The patient had no signs of infection or wound dehiscence for three weeks. The second stage was planned one month postoperatively, with total hip replacement augmented by a Burch-Schneider cage (Figure 5).

The cemented femoral stem of size 0 with a new 28 mm head was used with a cage of size 55 mm and augmented with an iliac crest bone graft over the acetabular side. Cage was used due to lysis and loss of bone in the medial wall with severe thinning. As the previous stem could be removed without ethylene oxide (EtO), the long stem could be avoided; the added advantage of the cemented stem is antibiotics in the cement.

**Figure 6:**
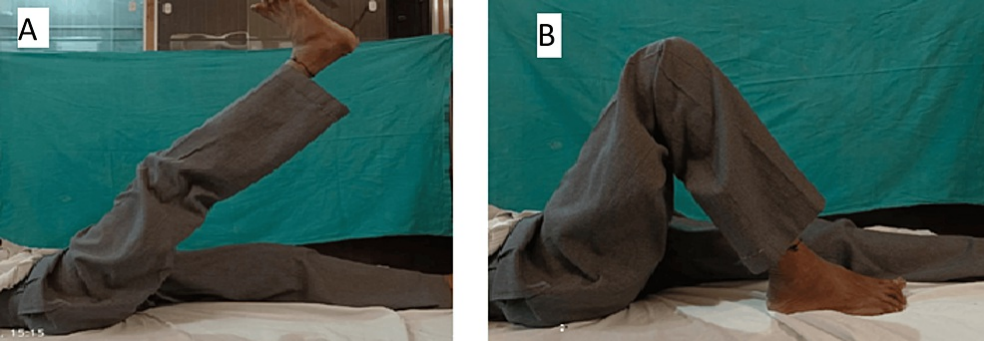
Follow-up range of motion at hip after three months (A) Range of motion with the knee extended. (B) Range of motion with the knee flexed.

Other surgical options were morselised and structural bone grafting with uncemented/cemented THR, and biological cages like a trabecular metal acetabular reconstruction cup cage construct can be used. Still, due to the patient's financial constraints and the non-availability of allograft, we have used the Burch-Schneider cage with cemented THR. Following surgery, the patient remained hospitalised for 12 days to monitor for early perioperative complications closely. Sutures were removed on the 12th day, indicating an absence of infection. Three months after the operation, the patient experienced notable improvements in pain levels, as well as in range of motion (ROM) and hip function (Figure 6).

## Discussion

Total hip arthroplasty (THA), frequently conducted during advanced osteoarthritis stages, is a medical intervention to reinstate mobility and alleviate the typical pain linked to arthritis, hip-related ailments, or injuries. Various elements influence the effectiveness and longevity of a hip replacement, encompassing device design specifics, surgeon expertise, implantation methodology, and individual patient traits like age, gender, body weight, activity levels, and overall health status. For MoM implants, the mechanism of contamination formation is trunk corrosion on the articular surface. However, for solutions other than MoM, contamination occurs at the interface of the modular neck post due to differences in the metal interface [2,3]. In this case study, MoP implants or non-MoM implants are the cause of ARMD. In more recent studies, the cause was also the lack of MoM [4-10].

Histopathological studies have revealed adverse local tissue reactions (ALTR) in both MoM and dual modular MoP system hip replacements, indicating similar origins and progression of these responses. There are differences in necrosis extension and ALTR. Thickness between MoM and MoP systems may stem from specific patient susceptibilities. Notably, a significantly lower incidence of ALTR occurs in non-modular MoP systems than in MoM systems [11]. This disparity might be attributed to milder symptomatology in MoP implants and lower clinician awareness, potentially allowing persistent damage in MoP, contrasting with heightened clinical suspicion and lower revision thresholds in MoM cases. While metal ions are typically analysed as controls in well-functioning MoP implants, recent studies suggest a 1 μg/L threshold for identifying ALTR in MoP hip implants [12]. Monitoring metal levels in patients' blood aids in predicting ALTR development in MoM hip arthroplasty and may prove useful in non-modular MoP systems. However, further research is necessary to establish clinically relevant thresholds for MoP implants distinct from dual-module MoM and MoP systems. Despite this, uncertainties persist regarding pre-operative management and ARMD diagnosis, leading to complete hip replacement failure. Surgical approaches for metal debris reactions in MoP hip replacements encompass diverse strategies like revision surgery, debridement and preservation, synovectomy, soft tissue repair, implant exchange or removal, tissue management, and material selection to mitigate complications and relieve symptoms.

Our clinical case affirms the absence of alerts about metal or serum ion levels concerning ARMD as serum and urinary cobalt (Co) and chromium (Cr) levels fell within standard limits pre-operatively. Additionally, our case underscores that among the primary complications of THA, soft tissue inflammatory reactions, collectively termed adverse reactions to metal debris (ARMD), contribute to implant failure, and a two-stage approach that we have taken in our case can be taken as a treatment option for long-standing ARMD in MoP hip system.

## Conclusions

ARMD in MoP hip implants underlines the importance of ongoing vigilance and research in orthopaedic surgery. Despite the lower incidence compared to metal-on-metal implants, ARMD in MoP implants poses significant challenges for patients and healthcare providers. Timely diagnosis and careful monitoring of patients with MoP implants are paramount. Regular follow-ups, including clinical evaluations and imaging studies, are essential to promptly detect any signs of adverse reactions. Additionally, a collaboration between orthopaedic surgeons, pathologists, and researchers is crucial to fully understanding the underlying mechanisms of ARMD in MoP implants.

## References

[1] Campbell J, Rajaee S, Brien E, Paiement GD (2017). Inflammatory pseudotumor after ceramic-on-ceramic total hip arthroplasty. Arthroplast Today.

[2] Bedard NA, Burnett RA, DeMik DE, Gao Y, Liu SS, Callaghan JJ (2017). Are trends in total hip arthroplasty bearing surface continuing to change? 2007-2015 usage in a large database cohort. J Arthroplasty.

[3] Langton DJ, Jameson SS, Joyce TJ, Hallab NJ, Natu S, Nargol AV (2010). Early failure of metal-on-metal bearings in hip resurfacing and large-diameter total hip replacement: a consequence of excess wear. J Bone Joint Surg Br.

[4] Barrett WP, Kindsfater KA, Lesko JP (2012). Large-diameter modular metal-on-metal total hip arthroplasty: incidence of revision for adverse reaction to metallic debris. J Arthroplasty.

[5] Reito A, Lainiala O, Elo P, Eskelinen A (2016). Prevalence of failure due to adverse reaction to metal debris in modern, medium and large diameter metal-on-metal hip replacements--the effect of novel screening methods: systematic review and metaregression analysis. PLoS One.

[6] Matharu GS, Pandit HG, Murray DW, Judge A (2016). Adverse reactions to metal debris occur with all types of hip replacement not just metal-on-metal hips: a retrospective observational study of 3340 revisions for adverse reactions to metal debris from the National Joint Registry for England, Wales, Northern Ireland and the Isle of Man. BMC Musculoskelet Disord.

[7] Vendittoli PA, Massé V, Kiss MO, Lusignan D, Lavigne M (2019). Modular junction may be more problematic than bearing wear in metal-on-metal total hip arthroplasty. Hip Int.

[8] Reito A, Elo P, Puolakka T, Pajamäki J, Eskelinen A (2015). Femoral diameter and stem type are independent risk factors for ARMD in the large-headed ASR THR group. BMC Musculoskelet Disord.

[9] Sassoon AA, Barrack RL (2016). Pseudotumour formation and subsequent resolution in metal-on-metal total hip arthroplasty following revision: instructional review and an illustrative case report with revision using a dual mobility design. Bone Joint J.

[10] Matharu GS, Berryman F, Dunlop DJ, Revell MP, Judge A, Murray DW, Pandit HG (2019). No threshold exists for recommending revision surgery in metal-on-metal hip arthroplasty patients with adverse reactions to metal debris: a retrospective cohort study of 346 revisions. J Arthroplasty.

[11] Natu S, Sidaginamale RP, Gandhi J, Langton DJ, Nargol AV (2012). Adverse reactions to metal debris: histopathological features of periprosthetic soft tissue reactions seen in association with failed metal on metal hip arthroplasties. J Clin Pathol.

[12] Fillingham YA, Della Valle CJ, Bohl DD, Kelly MP, Hall DJ, Pourzal R, Jacobs JJ (2017). Serum metal levels for diagnosis of adverse local tissue reactions secondary to corrosion in metal-on-polyethylene total hip arthroplasty. J Arthroplasty.

